# Rapid Detection of VOCs from Pocket Park Surfaces for Health Risk Monitoring Using SnO_2_/Nb_2_C Sensors

**DOI:** 10.3390/bios15070457

**Published:** 2025-07-15

**Authors:** Peng Wang, Yuhang Liu, Sheng Hu, Haoran Han, Liangchao Guo, Yan Xiao

**Affiliations:** 1 College of Mechanical Engineering, Yangzhou University, Yangzhou 225127, China; wp19980625@163.com (P.W.); liuyuhang@njust.edu.cn (Y.L.); husheng325945@163.com (S.H.); hhr200005@163.com (H.H.); glch2021@yzu.edu.cn (L.G.); 2 School of Architecture and Fine Art, Dalian University of Technology, Dalian 116024, China

**Keywords:** gas sensor, SnO_2_/Nb_2_CT_x_ MXenes, VOCs gas, health risk monitoring

## Abstract

The organic volatile compound gases (VOCs) emitted by the rubber running tracks in the park pose a threat to human health. Currently, the challenge lies in how to detect the VOC gas concentration to ensure it is below the level that is harmful to human health. This study developed a low-power acetone gas sensor based on SnO_2_/Nb_2_C MXene composites, designed for monitoring acetone gas in pocket park rubber tracks at room temperature. Nb_2_C MXene was combined with SnO_2_ nanoparticles through a hydrothermal method, and the results showed that the SnO_2_/Nb_2_C MXene composite sensor (SnM-2) exhibited a response value of 146.5% in detecting 1 ppm acetone gas, with a response time of 155 s and a recovery time of 295 s. This performance was significantly better than that of the pure SnO_2_ sensor, with a 6-fold increase in response value. Additionally, the sensor exhibits excellent selectivity against VOCs, such as ethanol, formaldehyde, and isopropanol, with good stability (~20 days) and reversibility (~50). It can accurately recognize acetone gas concentrations and has been successfully used to simulate rubber track environments and provide accurate acetone concentration data. This study provides a feasible solution for monitoring VOCs in rubber tracks and the foundation for the development of low-power, high-performance, and 2D MXene gas sensors.

## 1. Introduction

With the acceleration of urbanization, pocket parks, as scarce public spaces in high-density urban areas, have increased in number year by year. However, the release of volatile organic compounds (VOCs), including acetone, from plastic tracks under high temperatures poses a severe threat to children’s health. As a common additive in plastic materials, acetone can cause various health problems when exposed to high concentrations over long periods, including central nervous system depression, respiratory irritation, eye and skin damage, and even potential liver and kidney dysfunction [1]. Additionally, acetone is highly flammable, and when it reaches a certain concentration in the air, it can initiate explosions or fires, posing a potential risk to industrial safety and public spaces [2]. Therefore, monitoring and control of acetone release from plastic tracks were crucial for safeguarding public health.

In recent years, chemical resistance-type gas sensors have achieved advancements in acetone monitoring [3]. Zheng designed a Pt@In_2_O_3_ composite material with an eggshell-like structure based on metal oxide In_2_O_3_, achieving high-performance detection of acetone gas [4]. The material showed a response value of up to 57.1 for 100 ppm acetone at 300 °C, with response and recovery times of only 1 s and 44 s, respectively, and a detection limit as low as 0.5 ppm. Hu successfully achieved ppb-level acetone detection by preparing a porous Co_3_O_4_ nanosheet array derived from MOFs and assembling it onto SnO_2_ nanofibers [5]. The sensor showed a response value of 16.7 for acetone at 200 °C, with response and recovery times of 4 s and 39 s, respectively. Despite significant advancements in existing technologies, issues such as high working temperatures and insufficient selectivity still prevent them from meeting the application of outdoor park health monitoring.

SnO_2_ is a typical wide-bandgap (3.6 eV) n-type metal oxide semiconductor [6,7]. Due to its excellent chemical stability, low cost, and inherent sensitivity toward reducing gases, it became a fundamental material in gas sensing technology [8,9,10,11]. Its sensing mechanism primarily involves the adsorption-desorption process between target gas molecules and the oxygen species (O^−^, O_2_^−^) pre-adsorbed on the SnO_2_ surface. This process induces changes in carrier density through electron exchange, thereby leading to a variation in resistance. However, the performance of the original SnO_2_-based sensors was often affected by the severe agglomeration of nanoparticles, which decreases the available active sites and restricts gas diffusion pathways. The layered structure, tunable surface functionality, excellent conductivity, and high specific surface area of MXenes provided an ideal platform for anchoring SnO_2_ nanoparticles [12,13]. Specifically, MXenes exhibited a synergistic effect on the sensing performance due to their inherent gas response properties, showing enhanced selectivity to polar molecules. Wu developed a Ni_3_(HITP)_2_/Nb_2_C MXene heterostructure for ppb-level ethanol detection [14]. Zhou successfully used plasma-functionalized Nb_2_CT_x_ MXene for NH_3_ detection [15]. Moreover, MXenes can operate efficiently at room temperature, making them suitable for the development of low-power gas sensors and avoiding the energy consumption issues of traditional materials that require high-temperature heating [16,17,18,19,20].

In this work, we employed a heterojunction coupling strategy to develop new sensing materials for acetone monitoring. Using an improved hydrothermal synthesis method, SnO_2_ nanoparticles were successfully loaded onto Nb_2_C MXene nanosheets to prepare SnO_2_/Nb_2_C MXene composite materials. Experimental results showed that the SnO_2_/Nb_2_C MXene composite sensor (SnM-2) exhibited high response (146.5%) for 1 ppm acetone gas detection, with relatively fast response and recovery times (155/295 s), significantly outperforming pure SnO_2_-based sensors (response value was 6 times higher, response speed improved by 15%). This was attributed to the layered structure of MXenes, which mitigated the reduced effective surface area of SnO_2_ nanoparticles caused by aggregation, and the formed heterojunction significantly enhanced the overall performance of the composite material. Moreover, in simulated rubber track environment experiments, the sensor effectively detected acetone gas concentrations and successfully inferred the decay period of acetone gas in the rubber track (decaying to 1 ppm after 5 days), providing a feasible solution for gas release monitoring in sports field materials.

## 2. Materials and Methods

### 2.1. Materials

The pentahydrate tin chloride (SnCl_4_·5H_2_O), hydrazine hydrate (N_2_H_4_·H_2_O), 49% hydrofluoric acid (HF), and 20 wt% tetramethylammonium hydroxide (TMAOH) solution were all purchased from Shanghai McLin Biotech Co., Ltd. (Shanghai, China). The 400 mesh MAX phase (Nb_2_AlC) powder was purchased from Jilin Eleven Technology Co., Ltd. (Jilin, China). Deionized water was used throughout the experiments.

### 2.2. Synthesis of Nb_2_C MXene

Nb_2_C MXene was synthesized via HF etching of the Nb_2_AlC MAX phase precursor. In a PTFE beaker, 30 mL of 49% HF was added. Then, 2 g of Nb_2_AlC powder was slowly added to the beaker, with stirring at 500 rpm. The reaction was carried out at 50 °C for 72 h. After the reaction, the slurry was centrifuged and washed with deionized water multiple times until the pH of the supernatant reached 6–7. The resulting viscous precipitate was then added to 50 mL of 20 wt% TMAOH solution and stirred at room temperature for 24 h. After stirring, the precipitate was centrifuged twice at 8500 rpm with deionized water. Subsequently, the mixture was centrifuged at 3500 rpm for 30 min. Finally, the supernatant containing a few-layer Nb_2_C MXene was collected.

### 2.3. Synthesis of SnO_2_ Nanoparticles

Nanoparticles were synthesized via a hydrothermal method. First, 1.12 g of SnCl_4_·5H_2_O and 0.64 g of hydrazine hydrate (N_2_H_4_·H_2_O) were dissolved in 160 mL of deionized water. After stirring for 10 min, a white slurry formed, suggesting the formation of SnO_2_ intermediates during the reaction between N_2_H_4_ and SnCl_4_. This solution was then transferred to a 200 mL PTFE-lined autoclave. The autoclave was sealed and maintained at 120 °C for 6 h, then allowed to cool to room temperature naturally. Post-reaction, the solution was centrifuged and filtered. The resulting precipitate was washed with ethanol and deionized water, then dried in an oven at 60 °C for 1 h to obtain SnO_2_ nanoparticles.

### 2.4. Synthesis of SnO_2_/Nb_2_C MXene

Composite materials were prepared by mixing Nb_2_C MXene and SnO_2_ nanoparticles at different mass ratios to prepare the composite material. Specifically, three composite materials (SnM-1, SnM-2, and SnM-3) were obtained by mixing 100 mg of SnO_2_ nanoparticles with 10 mg, 20 mg, and 30 mg of Nb_2_C MXene, respectively. The mixture was ultrasonically dispersed in ethanol for 2 h. The mixture was then transferred to a hydrothermal reactor and reacted at 120 °C for 6 h to promote the integration of Nb_2_C MXene and SnO_2_ nanoparticles. After the reaction, the mixture was washed and dried to obtain the three SnM-*n* (*n* = 1, 2, 3) composites.

### 2.5. Preparation of Gas Sensors

The four composite materials, SnO_2_, SnM-1, SnM-2, and SnM-3, were each suspended in an ethanol solution. After ultrasonic dispersion, the suspension was drop-coated on the Pt electrode (the size of the Pt electrode was 2 mm × 2 mm) and then dried in an oven at 60 °C for 6 h to form the sensitive layer of the gas sensor. After the sensitive layer was dried, the sensor was completed and ready for testing.

### 2.6. Gas Sensitivity Testing

Gas sensitivity measurements were performed using an SD101 gas sensor testing device (Huachuang Ruike, China). The device consists of three parts: the gas sensitivity testing module, a static testing chamber, and data acquisition software. Two pairs of sensors coated with SnM-1, SnM-2, SnM-3, and SnO_2_ gas-sensitive materials were inserted into the slots of the gas sensitivity testing module. The gas channel leading to the static testing chamber was independently controlled by a mass flow controller (MFC). In this setup, the gas flow rate was precisely set to 200 sccm (standard cubic centimeters per minute), using pure air as the balancing gas. After the sensors stabilized in air, the target gas was introduced into the static testing chamber to evaluate the sensors’ gas sensitivity performance. During testing, the data acquisition software (HCRK-SD101 V2.3) displayed the time variation of the sensor’s resistance.

For the performance of the sensing material, the gas sensor response was defined as:

(1) $$\mathrm{Response}=\frac{\left(R_{a}-R_{g}\right)}{R_{g}}\;\times \;100\%$$

where R_a_ is the sensor resistance in air, and R_g_ is the resistance in the target gas. The time taken for the sensor to reach 90% of the total resistance change during the response and recovery phases is called the response time and recovery time, respectively.

### 2.7. Characterization Methods

To investigate the overall morphology of MXenes and composite materials, field emission scanning electron microscopy (FE-SEM, Hitachi S-4800, Hitachi High-Tech Corporation, Hitachi City, Japan) was used for morphological analysis, and EDS realizes micro-area component analysis through characteristic X-ray energy dispersion. This time, FE-SEM combined with an SDD detector (energy resolution < 125 eV) was used, configured with 20.0 kV acceleration voltage, 10 nA beam current, and 60 s/frame counting time, and the quantitative accuracy was ensured by Filter Fit fitting and the Proza correction model. Additionally, X-ray diffraction (XRD, D8 ADVANCE, Bruker Corporation, Karlsruhe, Germany) was employed to study the phase and crystal structure of the materials. To examine the band structure and surface state of the materials, X-ray photoelectron spectroscopy (XPS, Thermo Fisher Science, ESALAB 250Xi, Manchester, UK) was used. During the spectrum acquisition process, a constant analyzer energy mode was applied. For high-resolution scans of specific elements, the pass energy was set to 20 eV with a step size of 0.05 eV, while the pass energy for survey scans was 100 eV with a step size of 1 eV.

## 3. Results and Discussions

### 3.1. Morphology and Structural Characterization

**Figure 1:**
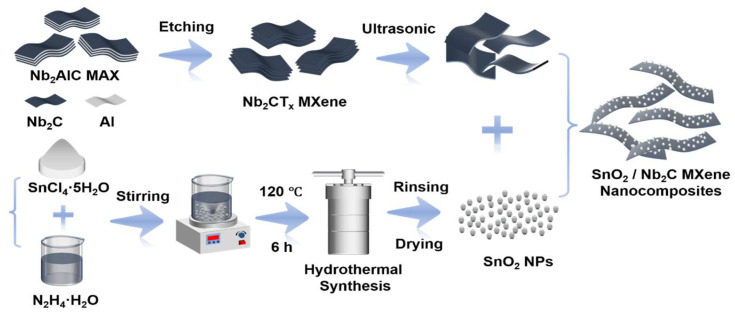
Schematic of SnO_2_/MXene synthesis process.

The synthesis process of the SnO_2_/MXene nanocomposite material is illustrated in Figure 1. The process first involved selectively etching Nb_2_AlC MAX phase to prepare highly conductive Nb_2_CT_x_ MXene nanosheets [21]. Then, under hydrothermal conditions, the MXene nanosheets were integrated with SnO_2_ precursors via a hydrothermal reaction. This stepwise synthesis strategy effectively constructed a hierarchical composite system of SnO_2_ nanoparticles and the MXene layered structure, thereby enhancing the interface contact between the two and providing a platform for charge transfer and gas molecule adsorption.

**Figure 2:**
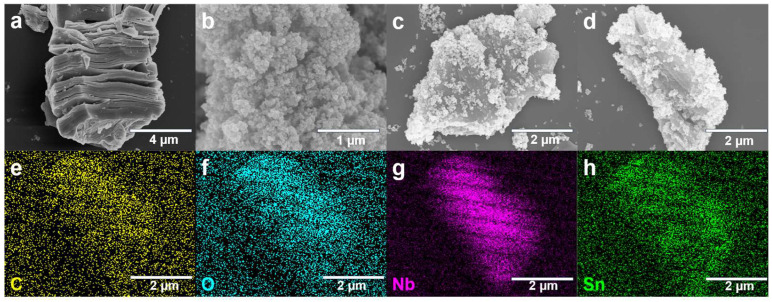
Characterization results. (**a**–**c**) Scanning electron microscope (SEM) images of (**a**). Nb_2_C MXene, (**b**). SnO_2_, (**c**). SnO_2_/MXene Nanocomposites. (**d**–**h**) Elemental mapping images of C, O, Nb, and Sn.

The scanning electron microscopy (SEM) images of the composite material’s morphology are shown in Figure 2a–c. Figure 2a shows that after hydrothermal treatment, Nb_2_C MXene exhibited a typical “accordion” layered structure, with thin nanosheets in a loosely stacked state [22]. This structure increased the material’s specific surface area, facilitating the diffusion and adsorption of gas molecules. Figure 2b indicates that SnO_2_ nanoparticles were approximately spherical, with a uniform particle size distribution, though mild aggregation of nanoparticles was observed. Figure 2c presents the microscopic morphology of the SnO_2_/MXene composite material: after the introduction of the Nb_2_CT_x_ MXene skeleton, some SnO_2_nanoparticles are anchored on the surface of the MXene nanosheets, effectively preventing the aggregation of SnO_2_, but based on the mass ratio of 100 mg SnO_2_ to 20 mg MXene, there are still a small amount of incompletely bound SnO_2_ free particles. This structural feature can effectively promote charge transfer and enhance the adsorption and reactive activity of gas molecules on the composite material’s surface.

Figure 2d–h illustrates the energy dispersive spectroscopy (EDS) element mapping analysis of the composite material. The elements of C and Nb were distributed uniformly within the MXene substrate region, while the elements of Sn and O were concentrated in the SnO_2_ nanoparticle area, indicating that SnO_2_ had been successfully loaded onto the MXene surface and was distributed evenly. The uniform distribution of all elements demonstrated the homogeneous distribution within the composite structure, a crucial element in ensuring the stability and repeatability of the gas sensor’s response.

**Figure 3:**
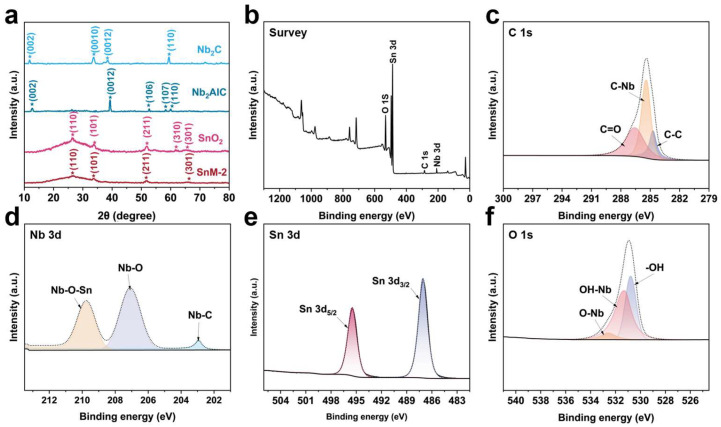
(**a**) XRD patterns of Nb_2_CTx MXene, Nb_2_AlC MAX, SnO_2_ and SnM-2, (**b**) XPS full survey spectrum; XPS profiles of (**c**) C 1s, (**d**) Nb 3d, (**e**) Sn 3d, and (**f**) O 1s.

Figure 3a shows the XRD patterns of Nb_2_CTx MXene, Nb_2_AlC MAX, SnO_2,_ and SnM-2 composites. For Nb_2_C MXene, clear diffraction peaks corresponding to the (002), (0010), (0012), and (110) planes were observed, respectively. The prominent and sharp (0010) peak indicates a high degree of crystallinity along the *c*-axis, which is a hallmark of its layered structure. The presence of the (002) peak further confirms the ordered stacking of the MXene layers, while the (110) peak appearing at a higher angle reflects the lattice constant along the *c*-axis and exhibits crystallographic anisotropy. In contrast, the Nb_2_AlC MAX phase exhibits a clear XRD pattern with peak positions that are significantly different from those of Nb_2_C MXene, indicating that the Al layer has been successfully etched and a MXene with an increased interlayer spacing has been formed. For the SnM-2 composite material composed of SnO_2_ and Nb_2_C MXene, the XRD pattern mainly shows the characteristic peaks of SnO_2_, such as (110), (101), (211), (310), and (301), which are consistent with its crystal structure. In addition, due to the low content of MXene in the composite sample, the diffraction peak of SnO_2_ is obvious, and the peak position of MXene is not well displayed [23,24].

To analyze the surface chemical states of the SnO_2_/MXene composite material, XPS characterization was conducted (Figure 3b–f). The survey spectrum (Figure 3b) shows the presence of elements such as Sn, Nb, O, and C, which correspond to the constituent elements of MXene (Nb, C) and SnO_2_ (Sn, O), indicating the successful synthesis of the SnO_2_/MXene composite [25]. The C 1s spectrum (Figure 3c), after multi-peak fitting, was decomposed into chemical states of C-Nb, C=O, and C-C. Among these, the C-Nb peak had a lower binding energy, indicating the formation of a strong chemical bond between the carbon and niobium atoms, which is a typical carbon–niobium bond in MXene materials. The appearance of the C=O peak could be related to oxidation or the adsorption of oxygen on the sample surface. The C-C peak corresponds to the carbon atoms between the MXene layers, reflecting the presence of carbon atoms in the layered structure of MXene. The Nb 3d spectrum (Figure 3d) revealed the chemical environments of Nb-C, Nb-O-Sn, and Nb-O. The Nb-C peak had a lower binding energy, indicating the formation of a stable chemical bond between niobium and carbon atoms, further confirming the presence of the MXene structure. The Nb-O-Sn peak indicated that there was an oxygen-mediated interaction between Nb and Sn, likely due to the composite formation between SnO_2_ and Nb_2_CT_x_, which resulted in the formation of Nb-O-Sn chemical bonds. The Nb-O peak corresponded to the oxidized state of niobium atoms, indicating that some niobium atoms had bonded with oxygen [26]. The Sn 3d spectrum (Figure 3e) consisted of two peaks, Sn3d_5/2_ and Sn3d_3/2,_ with a binding energy difference of approximately 8.4 eV between them, suggesting that the Sn element exists in a single chemical state, namely Sn^4+^ ions, which is consistent with the chemical state of Sn in SnO_2_, further confirming the presence of SnO_2_ and its chemical state in the composite material. The O 1s spectrum (Figure 3f), after fitting, identified functional groups such as O-Nb, OH-Nb, and -OH. The O-Nb peak corresponded to oxygen atoms bonded with niobium, reflecting the presence of oxygen atoms in Nb_2_CT_x_. The OH-Nb peak indicated the presence of niobium hydroxyl groups, possibly related to the hydroxylation of the MXene surface. The -OH peak likely corresponded to water molecules or hydroxyl free radicals adsorbed on the sample surface [27].

Based on the XRD and XPS analysis results, it can be inferred that there is a strong interaction between SnO_2_ and Nb_2_CT_x_ in the SnO_2_/Nb_2_CT_x_ MXene composite material. The XRD analysis confirmed the successful fabrication of the MXene layered structure. The XPS analysis revealed the presence of various chemical states and functional groups on the composite material’s surface, especially the interaction between SnO_2_ and Nb_2_CT_x_. This interaction, along with the rich functional groups on the material’s surface, significantly affects the gas-sensing performance of the composite material, providing a theoretical basis for further optimization of its properties.

In addition, the study provided further explanations for the XRD analysis and supplemented the Brunauer-Emmett-Teller (BET) specific surface area test to enhance the characterization of the material structure. The relevant experimental data and patterns are presented in Figures S1 and S2 of the Supporting Information.

### 3.2. Performance of Gas Sensors

**Figure 4:**
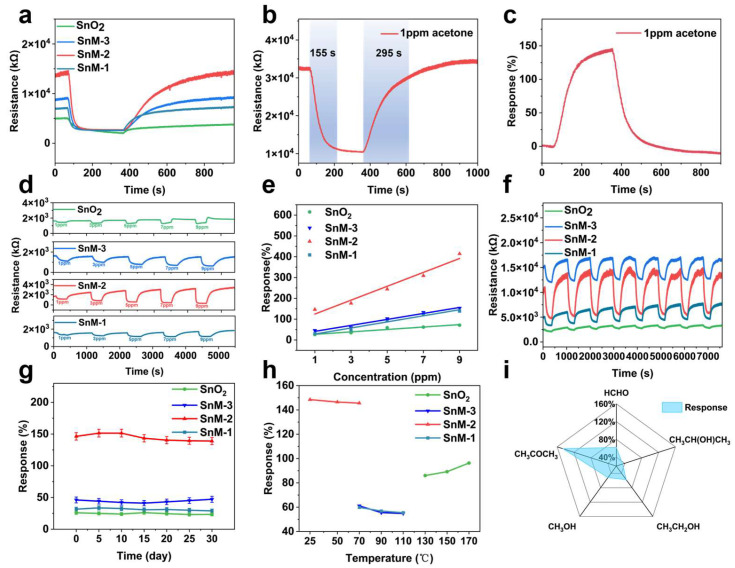
(**a**) The response–time (R–T) curves of four sensors exposed to 1 ppm acetone gas; (**b**) the response and recovery time profile of the SnM-2 sensor to 1 ppm acetone gas; (**c**) the dynamic response curve of the SnM-2 sensor over time; (**d**) the cyclic stability of the four sensors upon exposure to 1 ppm acetone gas; (**e**) the R–T curves of the four sensors exposed to acetone gas concentrations ranging from 1 to 9 ppm; (**f**) the linear relationship between sensor response and acetone concentration; (**g**) the long-term stability of the four sensors; (**h**) the optimal operating temperature range for the four sensors; (**i**) the selectivity of the SnM-2 sensor toward acetone gas.

In this study, the response intensity of SnO_2_, SnM-1, SnM-2, and SnM-3 sensors to 1 ppm acetone at room temperature was evaluated. Figure 4a presents the dynamic sensor response transients of the four sensors to 1 ppm acetone at room temperature. The response values of the SnM-*n* (*n* = 1, 2, 3) sensors were considerably higher than those of the SnO_2_ sensor, indicating that the gas-sensing performance of the composite material was enhanced after the introduction of the MXene framework. Concurrently, the SnM-2 sensor demonstrated the most abbreviated response time (approximately 155 s) and recovery time (295 s, Figure 4b), accompanied by a response value of 146.5% (Figure 4c). Conversely, the response times of SnO_2_, SnM-1, and SnM-3 were prolonged to 180, 200, and 210 s, respectively, accompanied by corresponding increases in recovery times to 310, 320, and 330 s. This discrepancy suggested that the SnM-2 sensor exhibited superior dynamic response characteristics compared to the other sensors.

Furthermore, the response–recovery curves of the four sensors to 1–9 ppm acetone gas and the relationship between response values and concentration were examined (Figure 4d,e). As the acetone concentration increased, the response values of all sensors increased linearly, and the SnM-*n* (*n* = 1, 2, 3) series sensors showed a faster rise compared to the pure SnO_2_ sensor, indicating superior performance in acetone detection. Among the sensors examined, the SnM-2 sensor demonstrated the most substantial change in response, suggesting an increased gas sensitivity. The response to this stimulus was contingent upon the concentration of acetone and was the result of a combined effect of SnO_2_ and MXene. The stability of SnO_2_ served as a foundation for sensing applications, while the incorporation of MXene dopants enhanced the material’s overall performance. This phenomenon has been demonstrated to increase the number of active sites and enhance the electron transport path. Appropriate amounts of MXene mitigated the issues associated with excessive amounts of the material, which had been shown to prolong electron transport pathways. This equilibrium guaranteed optimal responsiveness and amplified the sensor’s signal to the acetone concentration.

We tested the cyclic stability of the four sensors to see if they could work well in real applications (Figure 4f). In multiple cycles of testing with 1 ppm acetone gas, the sensors demonstrated stable performance with no significant decay. The mean response values were 24.58%, 44.16%, 144.53%, and 31.21%, respectively. To assess the long-term stability of the sensors, they were exposed to 1 ppm acetone at room temperature for a period of 30 days (Figure 4g). After 30 days, the response values of the sensors demonstrated no significant decay. These findings indicated that the sensors exhibited exceptional long-term stability and longevity. The enhanced performance could be attributed to the stable effect of SnO_2_ and the superior electron transport properties of MXene. As illustrated in Figure 4h,i, these sensors demonstrated optimal temperature ranges and selectivity outcomes. The SnM-2 sensor demonstrated the highest response values from 25 °C to 50 °C, indicating its optimal performance for acetone detection within this temperature range. The SnM-2 sensor demonstrated notable selectivity for acetone gas. Notably, the response value of the sensor was found to be significantly higher than that of other gases, including ethanol, formaldehyde, and isopropanol, under conditions involving complex gas environments. This observation underscored the sensor’s notable resistance to interference from diverse gas compositions. Meanwhile, compared with other acetone gas sensors listed in Table 1, our SnM-2 sensor has many advantages in detecting acetone, especially in terms of sensitivity and low actual detection concentration.

Humidity is also an important factor affecting the gas-sensitive response. For this reason, we evaluated the effect of relative humidity (RH) levels from 20% to 80% on the sensing performance of 10 ppm acetone at room temperature. Figure S3 shows the gas-sensitive responses of SnO_2_, SnM-3, SnM-2, and SnM-1 sensors at different RH levels. As the RH increases, the gas-sensitive response values of all sensors show a downward trend, which is mainly due to the competitive adsorption of water molecules and acetone gas molecules on the active sites on the surface of the material. Specifically, at 20% relative humidity, the SnM-2 material exhibits the highest response value (about 412%), while the response value of SnO_2_ is the lowest (about 75%). It is worth noting that the SnM-2 sensor exhibits good moisture resistance, and the response value remains at 340% at 80% RH, which is much higher than the 60% of the pure SnO_2_ sensor. The results show that after the introduction of the Nb_2_CT_x_ MXene skeleton, the SnM-2 sensor can maintain good gas-sensing properties even in a high-humidity environment [20].

In summary, the SnO_2_ nanoparticle gas sensor modified with Nb_2_C MXene exhibited good performance in acetone gas detection and can be used for practical environmental monitoring.

### 3.3. Application

**Figure 5:**
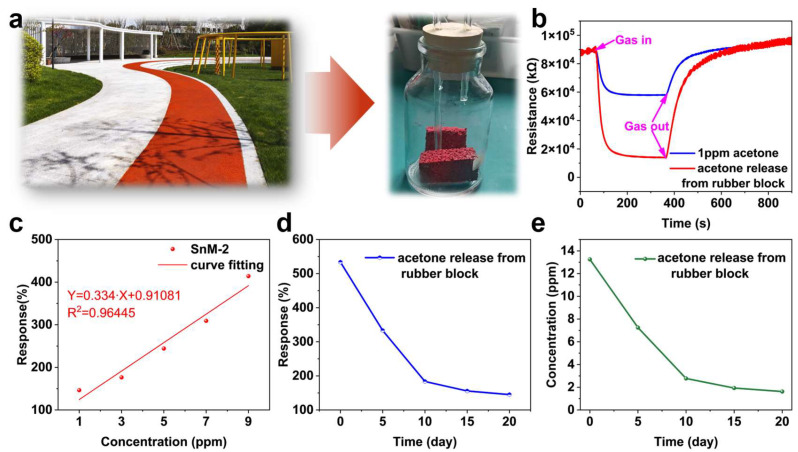
(**a**) Rubber block samples and testing bottles; (**b**) response curves of SnM-2 sensor to 1 ppm acetone gas and acetone gas released from rubber block samples; (**c**) response values of SnM-2 sensor to acetone gas at different concentrations and its linear fitting curve; (**d**) response values of acetone gas released by rubber block samples over 20 days; (**e**) concentration variation curve of acetone gas released by rubber blocks over 20 days (calculated based on the linear fitting curve).

To systematically evaluate the volatilization characteristics of acetone in rubber tracks, this study conducted simulation experiments using newly prepared rubber blocks (Figure 5a). The experimental materials used were red granular rubber samples, which were processed and placed in a custom-sealed container to replicate the slow-release process of acetone under real-world conditions [34,35]. The variation in acetone release was monitored using the SnM-2 sensor. The SnM-2 sensor continuously monitored the acetone gas released from the rubber block, recording the sensor’s resistance changes, which were then converted into acetone concentration values to quantitatively assess the acetone release.

Figure 5b presents the response curves of the sensor to 1 ppm of acetone gas and acetone gas released from the rubber block. It can be observed that the resistance curve of acetone gas from the newly prepared rubber block exhibits significant variation. This not only indicates that the acetone content in the newly prepared rubber block is relatively high but also demonstrates the applicability of the SnM-2 sensor in environmental monitoring. Figure 5c shows the response values of the SnM-2 sensor to different concentrations of acetone gas and their linear fit line. The experimental results indicate a good linear relationship between the sensor’s response values and acetone concentration, with the linear equation Y = 0.334·X + 0.91081 and R^2^ = 0.96445. This suggests that the SnM-2 sensor has excellent linearity and quantitative analysis capabilities within its detection range, enabling it to accurately reflect changes in acetone gas concentration and enable quantitative assessment of the acetone release from the rubber block.

Additionally, we tracked the variation in response values of the rubber block sample releasing acetone gas over 20 days (Figure 5d). The results indicated that the acetone release exhibited a three-stage decay characteristic: the initial stage (0–5 days) showed a rapid release rate, the middle stage (5–15 days) exhibited a slower decay, and the final stage (15–20 days) reached a stable level. Subsequently, based on the linear response characteristics of the SnM-2 sensor, we inverted the data to calculate the acetone gas concentration change curve over the 20 days (Figure 5e). The calculation results showed that the rubber block initially released a higher concentration, which gradually decreased over time. This trend was consistent with the response value changes observed in Figure 5c, further verifying the reliability of the sensor data. By monitoring the release behavior of acetone gas, this approach provides a theoretical basis for assessing the acetone release from newly constructed rubber tracks and formulating appropriate control measures.

### 3.4. Gas Sensing Mechanism

**Figure 6:**
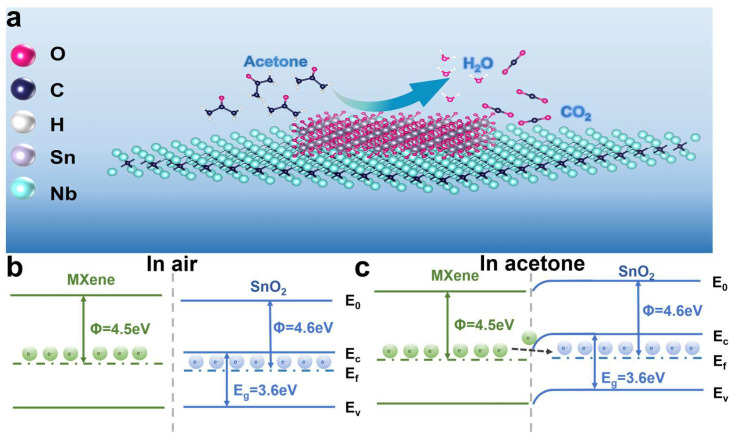
(**a**) Schematic diagram of gas-sensing mechanism and (**b**,**c**) band diagram of SnO_2_/MXene nanocomposites.

In this study, the sensing mechanism of the SnM-2 sensor for acetone gas was based on the sensing principle of n-type metal oxide semiconductor materials, which mainly involved the adsorption of gas molecules on the material’s surface, redox reactions, and electron transport processes (Figure 6). In air, oxygen molecules adsorbed onto the surface of the SnM-2 sensor and captured electrons from the conduction band of SnO_2_, forming oxygen ions (O^−^, O_2_^−^), as shown in Equations (2)–(4):

(2) $$O_{2(\mathrm{gas})}\to O_{2(\mathrm{ads})}$$

(3) $$O_{2(\mathrm{gas})}+e^{-}\to O_{2(\mathrm{ads})}^{-}$$

(4) $$O_{2(\mathrm{ads})}^{-}+e^{-}\to 2O_{\left(\mathrm{ads}\right)}^{-}\;$$

These oxygen ions form an electron-depleted layer on the material’s surface, leading to an increase in the sensor’s resistance [36,37]. When the SnM-2 sensor was exposed to acetone gas, the adsorbed oxygen ions underwent redox reactions with acetone molecules, as shown in Equation (5):

(5) $${\mathrm{CH}}_{3}\mathrm{CO}{\mathrm{CH}}_{3(\mathrm{gas})}+8O_{\left(\mathrm{ads}\right)}^{-}\to 3{\mathrm{CO}}_{2(\mathrm{gas})}+3H_{2}O_{\left(\mathrm{gas}\right)}+8e^{-}$$

In this reaction process, acetone molecules were oxidized, releasing electrons back into the conduction band of SnO_2_. The injection of these electrons reduced the electron-depleted layer, leading to a decrease in the sensor’s resistance. When the sensor was exposed to air again, oxygen molecules re-adsorbed onto the material’s surface, and the sensor’s resistance gradually returned to its original value.

The excellent sensing performance of the SnM-2 sensor can be attributed to three main factors. First, the introduction of the Nb_2_C MXene framework increased the material’s specific surface area and effectively prevented the aggregation of SnO_2_ nanoparticles, thereby exposing more active sites [38]. These active sites provided more adsorption space for gas molecules and facilitated the desorption process of the gas molecules. Additionally, the rich functional groups on the MXene surface (such as -O, -OH, and -F) served as adsorption sites, further enhancing the tunability and variability of surface electrons through hydrogen bonding or electronic interactions, thus improving the sensing response. These functional groups interacted with acetone gas molecules, enhancing the adsorption and desorption processes of acetone molecules on the material’s surface, thereby increasing the sensor’s sensitivity to acetone [39,40].

Second, the heterojunction interface formed between MXene and SnO_2_ plays a crucial role in enhancing the sensing performance. The heterojunction effect at the SnO_2_ and MXene interface leads to electron accumulation and depletion, forming a Schottky barrier. Specifically, due to the work function difference (SnO_2_: 4.6 eV; Nb_2_C MXene: 4.5 eV), electrons migrate from the lower work function MXene to the higher work function SnO_2_, forming a Schottky barrier at the interface that enhances charge separation and transport in Figure 6b,c [41,42]. The electron distribution at this interface undergoes significant changes, creating a charge-depleted region and forming the Schottky barrier. When the sensor is exposed to acetone gas, acetone molecules react with the oxygen species on the sensor surface, releasing the captured electrons. These electrons quickly enter the conduction band, leading to a significant decrease in the material’s resistance and a considerable increase in the response speed. After gas desorption, the released electrons quickly return to the interface, causing the resistance to rise and the recovery speed to improve significantly [43,44]. This mechanism allows the heterojunction structure between SnO_2_ and MXene to significantly accelerate both the response and recovery speeds [45,46,47].

Finally, the electronic transport path was optimized by appropriately doping MXene, preventing excessive MXene from causing an overextension of the electron transport path, which could affect efficient electron transfer. By properly controlling the amount of MXene doping, the optimal response speed and sensing performance could be ensured. In summary, these factors synergistically contributed to enhancing the acetone gas sensing performance of the SnO_2_/Nb_2_C MXene sensor.

## 4. Conclusions

This study successfully developed a gas-sensing material based on SnO_2_/Nb_2_C MXene heterojunction, enabling low-power environmental monitoring of pocket park rubber tracks. Through the strategic integration of MXene nanosheets, a unique heterojunction interface structure was constructed within the composite material. This structure not only significantly enhances the density of active sites on the material’s surface but also optimizes the electron transport path. Compared to pure SnO_2_ sensors, the SnM-2 composite sensor exhibited a 146.5% response value for 1 ppm acetone detection, with response/recovery times reduced to 155 s/295 s, a 6-fold increase in sensitivity, and good linear response to acetone gas at different concentrations. Furthermore, the sensor demonstrated reliable performance in simulated rubber track environment experiments and successfully inferred a decay period of 5 days to reach 1 ppm, providing a feasible solution for gas release risk assessment of sports field materials.

## Figures and Tables

**Table 1:** Properties comparison of acetone sensors.

Materials	Temperature	Concentration	Response	Res.	Ref.
WO_3_/CeO_2_	250 °C	2.5 ppm	1.7 ^a^	34 s	[28]
SnO_2_/rGO	RT	10 ppm	21.9% ^b^	107 s	[29]
ZnO/IGO	300 °C	100 ppm	27.1 ^a^	6.8 s	[30]
Ti_3_C_2_ MXene	RT	10 ppm	0.6% ^b^	45 s	[31]
Na-doped ZnO	RT	0.2 ppm	6.55 ^a^	18 s	[32]
SnO_2_/Fe_2_O_3_	270 °C	200 ppm	9.3 ^a^	6 s	[33]
SnO_2_/Nb_2_CT_x_	RT	1 ppm	146.5% ^b^	155 s	This work

^a^ The response was calculated by R_a_/R_g_ or R_g_/R_a_. ^b^ The response was calculated by ΔR/R_0_ × 100%.

## Data Availability

Data availability All data included in this study are available upon request by contact with the corresponding author.

## Supplementary Materials

The following supporting information can be downloaded at: https://www.mdpi.com/article/10.3390/bios15070457/s1, Figure S1: XRD patterns of Nb2CTx MXene, Nb2AlC MAX, SnO2, and SnM-2; Figure S2: (a) Nitrogen isotherms and (b) pore size distribution curves of SnO2 and SnM-2; Figure S3: The relationship between response values and RH, measured at 10 ppm acetone, under a humidity range of 20–80% RH; Table S1. Properties comparison of acetone sensors.
