# Supplementary material for: Rapid Detection of VOCs from Pocket Park Surfaces for Health Risk Monitoring Using SnO2/Nb2C Sensors
Source: Biosensors (Basel). 2025 Jul 15;15(7):457. doi: 10.3390/bios15070457 (PMC12293254; doi:10.3390/bios15070457)
Supplement: Supplementary file 1 [file biosensors-15-00457-s001.zip › biosensors-3702037-supplementary.pdf]

Supporting Information

Rapid Detection of VOCs from Pocket Park Surfaces for Health Risk Monitoring Using SnO_2/Nb_2C Sensors

Peng Wang [1], Yuhang Liu [1], Sheng Hu [1], Haoran Han [1], Liangchao Guo [1] and Yan Xiao [2,*]

1 College of Mechanical Engineering, Yangzhou University, Yangzhou 225127, China; wp19980625@163.com (P.W.); liuyuhang@njust.edu.cn (Y.L.); husheng325945@163.com (S.H.); hhr200005@163.com (H.H.); glch2021@yzu.edu.cn (L.G.)
2 School of Architecture and Fine Art, Dalian University of Technology, Dalian 116024, China
* Correspondence: xiaoyan@dlut.edu.cn

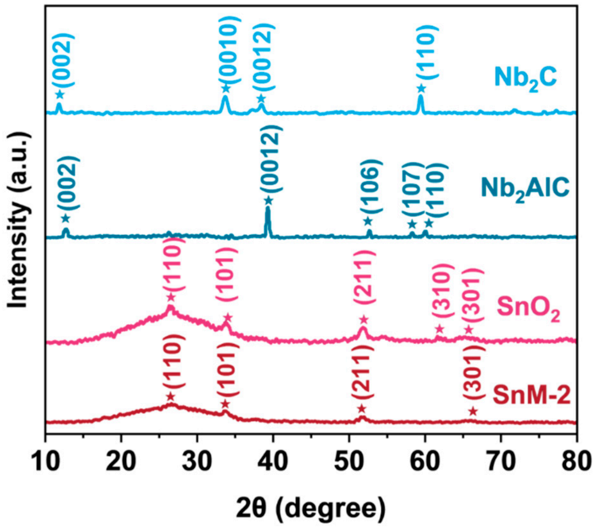

Figure S1. XRD patterns of Nb_2CTx MXene, Nb_2AlC MAX, SnO_2, and SnM-2.

Figure S1 shows the XRD patterns of Nb_2CT_x MXene, Nb_2AlC MAX, SnO_2, and SnM-2 composites. For Nb_2C MXene, clear diffraction peaks corresponding to the (002), (0010), (0012), and (110) planes were observed, respectively. The prominent and sharp (0010) peak indicates a high degree of crystallinity along the c-axis, which is a hallmark of its layered structure. The presence of the (002) peak further confirms the ordered stacking of the MXene layers, while the (110) peak appearing at a higher angle reflects the lattice constant along the c-axis and exhibits crystallographic anisotropy. In contrast, the Nb_2AlC MAX phase exhibits a clear XRD pattern with peak positions that are significantly different from those of Nb_2C MXene, indicating that the Al layer has been successfully etched and an MXene with an increased interlayer spacing has been formed. For the SnM-2 composite material composed of SnO_2 and Nb_2C MXene, the XRD pattern mainly shows the characteristic peaks of SnO_2, such as (110), (101),

(211), (310), and (301), which is consistent with its crystal structure. In addition, due to the low content of MXene in the composite sample, the diffraction peak of SnO_2 is obvious, and the peak position of MXene is not well displayed.

The BET (Brunauer–Emmett–Teller) specific surface area and pore size distribution of the synthesized samples were determined by nitrogen adsorption experiments. The results in Figure S2 show that the specific surface

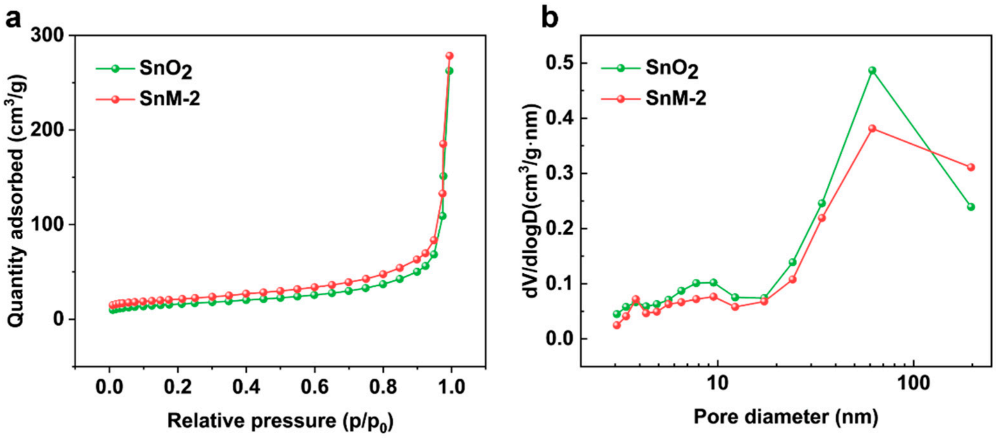

Figure S2. (a) Nitrogen isotherms and (b) pore size distribution curves of SnO_2 and SnM-2.

area of the SnO_2 sample is 54.656 m^2/g and the total pore volume is 0.4215 cc/g, indicating that it has a high porosity. In comparison, the specific surface area of the SnM-2 composite material is slightly increased to 57.220 m^2/g, which is 4.2% higher than that of pure SnO_2, and the total pore volume is 0.4062 cc/g, indicating that after being composited with MXene, the material still maintains a significant pore structure. In addition, the pore size distribution of the synthesized samples is mainly concentrated in the range of 40–80 nm. Overall, these favorable pore properties provide abundant reaction sites and diffusion channels for gas molecules, thereby effectively improving the gas-sensing properties of the material.

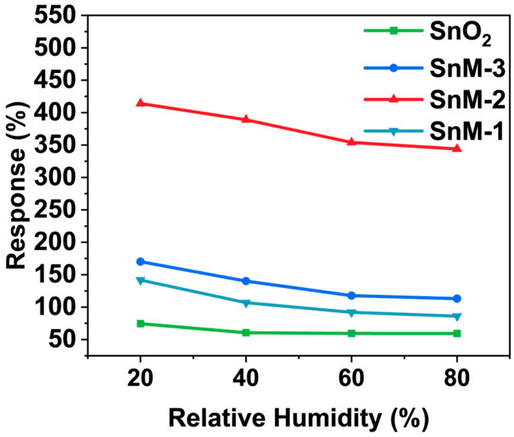

Figure S3. The relationship between response values and RH, measured at 10 ppm acetone, under a humidity range of 20– 80 % RH.

Humidity is also an important factor affecting the gas-sensitive response. For this reason, we evaluated the effect of relative humidity (RH) levels from 20% to 80% on the sensing performance of 10 ppm acetone at room temperature. Figure S3 shows the gas-sensitive responses of SnO_2, SnM-3, SnM-2, and SnM-1 sensors at different RH levels. As the RH increases, the gas-sensitive response values of all sensors show a downward trend, which is mainly due to the competitive adsorption of water molecules and acetone gas molecules on the active sites on the surface of the material. Specifically, at 20% relative humidity, the SnM-2 material exhibits the highest response value (about 412%), while the response value of SnO_2 is the lowest (about 75%). It is worth noting that the SnM-2 sensor exhibits good moisture resistance, and the response value remains at 340% at 80% RH, which is much higher than the 60% of the pure SnO_2 sensor. The results show that after the introduction of the Nb_2CT_x MXene skeleton, the SnM-2 sensor can maintain good gas-sensing properties even in a high humidity environment (20%).

Table S1. Properties comparison of acetone sensors.

Materials	Temperature	Concentration	Response	Res.	Ref.
WO_3/CeO_2	250 °C	2.5 ppm	1.7[a]	34 s	[28]
SnO_2/rGO	RT	10 ppm	21.9%[b]	107 s	[29]
ZnO/IGO	300 °C	100 ppm	27.1[a]	6.8 s	[30]
Ti_3C_2 MXene	RT	10 ppm	0.6%[b]	45 s	[31]

Na doped ZnO	RT	0.2 ppm	6.55 [a]	18 s	[32]
SnO_2/Fe_2O_3	270 °C	200 ppm	9.3 [a]	6 s	[33]
SnO_2/Nb_2CT_x	RT	1ppm	146.5% [b]	155 s	This work

a. The response was calculated by Ra/Rg or Rg/Ra.

b. The response was calculated by $\Delta R/R_0 \times 100$ %.

References

Yuan, K.; Wang, C.-Y.; Zhu, L.-Y.; Cao, Q.; Yang, J.-H.; Li, X.-X.; Huang, W.; Wang, Y.-Y.; Lu, H.-L.; Zhang, D.W. Fabrication of a Micro-Electromechanical System-Based Acetone Gas Sensor Using CeO_2 Nanodot-Decorated WO_3 Nanowires. *ACS Appl. Mater. Interfaces* **2020**, *12*, 14095–14104, doi:10.1021/acsami.9b18863.

Zhang, D.; Liu, A.; Chang, H.; Xia, B. Room-Temperature High-Performance Acetone Gas Sensor Based on Hydrothermal Synthesized SnO2-Reduced Graphene Oxide Hybrid Composite. *RSC Adv.* **2014**, *5*, 3016–3022, doi:10.1039/C4RA10942B.

Zhang, Y.; Jia, C.; Kong, Q.; Fan, N.; Chen, G.; Guan, H.; Dong, C. ZnO-Decorated In/Ga Oxide Nanotubes Derived from Bimetallic In/Ga MOFs for Fast Acetone Detection with High Sensitivity and Selectivity. *ACS Appl. Mater. Interfaces* **2020**, *12*, 26161–26169, doi:10.1021/acsami.0c04580.

Wu, M.; He, M.; Hu, Q.; Wu, Q.; Sun, G.; Xie, L.; Zhang, Z.; Zhu, Z.; Zhou, A. Ti3C2 MXene-Based Sensors with High Selectivity for NH3 Detection at Room Temperature. *ACS Sens.* **2019**, *4*, 2763–2770, doi:10.1021/acssensors.9b01308.

Jaisutti, R.; Lee, M.; Kim, J.; Choi, S.; Ha, T.-J.; Kim, J.; Kim, H.; Park, S.K.; Kim, Y.-H. Ultrasensitive Room-Temperature Operable Gas Sensors Using p-Type Na:ZnO Nanoflowers for Diabetes Detection. *ACS Appl. Mater. Interfaces* **2017**, *9*, 8796–8804, doi:10.1021/acsami.7b00673.

Yang, J.; Liu, J.; Xu, Y.; Li, X.; Wu, J.; Han, Y.; Wang, Z.; Zhang, X. Enhanced Selective Acetone-Sensing Performance of Hierarchical Hollow SnO2/α-Fe2O3 Microcubes. *J. Mater. Chem. C* **2019**, *7*, 11984–11990, doi:10.1039/C9TC03879E.
